# Neck cooling induces blood pressure increase and peripheral vasoconstriction in healthy persons

**DOI:** 10.1007/s10072-020-04349-x

**Published:** 2020-03-26

**Authors:** Julia Koehn, Ruihao Wang, Carmen de Rojas Leal, Bernd Kallmünzer, Klemens Winder, Martin Köhrmann, Rainer Kollmar, Stefan Schwab, Max J. Hilz

**Affiliations:** 1 Department of Neurology, University of Erlangen-Nuremberg, Schwabachanlage 6, 91054 Erlangen, Germany; 2 Department of Neurology, Universitätsklinikum Essen, Hufelandstr. 55, 45147 Essen, Germany; 3 Department of Neurology, General Hospital Darmstadt, Grafenstr. 9, 64283 Darmstadt, Germany; 4 Department of Neurology, Icahn School of Medicine at Mount Sinai, New York, NY USA

**Keywords:** Neck cooling, Hypothermia, Cerebral blood flow velocity, Blood pressure increase

## Abstract

**Introduction:**

Noninvasive temperature modulation by localized neck cooling might be desirable in the prehospital phase of acute hypoxic brain injuries. While combined head and neck cooling induces significant discomfort, peripheral vasoconstriction, and blood pressure increase, localized neck cooling more selectively targets blood vessels that supply the brain, spares thermal receptors of the face and skull, and might therefore cause less discomfort cardiovascular side effects compared to head- and neck cooling. The purpose of this study is to assess the effects of noninvasive selective neck cooling on cardiovascular parameters and cerebral blood flow velocity (CBFV).

**Methods:**

Eleven healthy persons (6 women, mean age 42 ± 11 years) underwent 90 min of localized dorsal and frontal neck cooling (EMCOOLS Brain.Pad™) without sedation. Before and after cooling onset, and after every 10 min of cooling, we determined rectal, tympanic, and neck skin temperatures. Before and after cooling onset, after 60- and 90-min cooling, we monitored RR intervals (RRI), systolic, diastolic blood pressures (BPsys, BPdia), laser Doppler skin blood flow (SBF) at the index finger pulp, and CBFV at the proximal middle cerebral artery (MCA). We compared values before and during cooling by analysis of variance for repeated measurements with post hoc analysis (significance: *p* < 0.05).

**Results:**

Neck skin temperature dropped significantly by 9.2 ± 4.5 °C (minimum after 40 min), while tympanic temperature decreased by only 0.8 ± 0.4 °C (minimum after 50 min), and rectal temperature by only 0.2 ± 0.3 °C (minimum after 60 min of cooling). Index finger SBF decreased (by 83.4 ± 126.0 PU), BPsys and BPdia increased (by 11.2 ± 13.1 mmHg and 8.0 ± 10.1 mmHg), and heart rate slowed significantly while MCA-CBFV remained unchanged during cooling.

**Conclusions:**

While localized neck cooling prominently lowered neck skin temperature, it had little effect on tympanic temperature but significantly increased BP which may have detrimental effects in patients with acute brain injuries.

## Introduction

Therapeutic hypothermia may have neuroprotective effects in patients with ischemic tissue injury, such as stroke and cardiac arrest [1]. Suggested beneficial effects of brain hypothermia are reduced oxygen demand and mitigated inflammatory mechanisms which may result in a decrease of cerebral edema and intracranial pressure [1].

In recent years, multiple different methods have been introduced to achieve brain hypothermia [2–4]. Endovascular temperature management has been demonstrated to induce brain hypothermia with the advantage of maintaining consistent target temperatures and the ability to control rewarming rates [2–4]. Yet, the method is invasive and associated with general, i.e., whole-body hypothermia and thus might cause systemic complications, e.g., coagulopathy, impaired immune function, pneumonia, or electrolyte disturbances [2–4]. Surface cooling has been demonstrated to be effective but less invasive [2–4]. Yet, surface cooling methods imply the disadvantage of temperature gradients between cortical and deeper brain areas due to heat exchange through the different layers of the cranium [2–4]. While neuroprotective effects seem to vary with different depths of hypothermia [5], even mild hypothermia induced by noninvasive surface cooling might be neuroprotective and therefore useful in the prehospital treatment of non-sedated stroke patients [1]. In healthy persons, Kallmünzer et al. showed that combined external head and neck cooling slightly reduces body core temperature, and suggested that the method might be useful for noninvasive, prehospital hypothermia induction [6]. Using the same method, we also observed a significant drop in skin temperature [7]. However, combined head and neck cooling also caused peripheral vasoconstriction and a prominent blood pressure (BP) increase by 15.3 ± 20.8 mmHg in healthy participants, while heart rates (HR) slowed by 6.5 bpm [7]. These responses are similar to those of the so-called cold face test [8, 9] where cold stimulation of the face induces peripheral sympathetic activation with vasoconstriction and BP increase and simultaneous cardiovagal activation with subsequent HR slowing [8]. The cold face test also alters cerebral blood flow (CBF) [9].

While head cooling requires cold conduction from the scalp to the brain [1] which may be rather limited due to the thermal barrier of the scalp and skull [1], isolated neck cooling might have the advantage of more directly cooling the carotid and vertebral arteries and thus intracranial blood which should yield the above hypothermia benefits [1].

Moreover, isolated neck cooling may be less uncomfortable than combined neck and head cooling since head cooling affects the skull and causes prominent deep pain [10]. In contrast, neck cooling may have more effect on blood vessels and soft tissue and less effect on boney structures and might therefore induce less deep pain than head cooling [10]. Effects of isolated neck cooling on cerebral blood flow velocities (CBFV) are unknown.

In order to evaluate the above effects, we assessed changes in HR, BP, and middle cerebral artery (MCA) CBFV and rated subjective discomfort and frostiness in response to isolated neck cooling in young healthy persons.

## Materials and methods

Eleven healthy volunteers (6 women, 5 men, mean age 42 ± 11 years) participated in the study. None of the participants had any known disease or was taking medication known to affect the cardiovascular or autonomic system. Before testing, all participants refrained from nicotine, caffeine, or alcohol for at least 18 h. The Institutional Ethics Committee of the University of Erlangen-Nuremberg had approved the study, and written informed consent had been obtained from all study participants according to the Declaration of Helsinki.

### Baseline recordings at supine rest and recordings during neck cooling

Participants were tested between 9 a.m. and 2 p.m. They were lying on a comfortable stretcher in a quiet room with an ambient temperature of 24 °C and stable humidity. All participants initially rested for 40 min to ensure a stable cardiovascular situation while we attached the monitoring devices.

Cold stimulation was accomplished with a neck cooling device (EMCOOLS Brain.Pad™) containing HypoCarbon cooling gel® and consisting of two cooling elements for the neck and two cooling elements for the shoulders. The cooling device is layered with a skin-friendly and dermatologically tested medical adhesive film and sticks onto the participant’s skin until removed. Before use, the cooling device was kept at 4 °C [11].

Temperature was measured with one skin probe attached to the neck (Bio Thermostat BTH-5, s.i.r., Germany), another probe inserted into the rectum (Temprecise, Arizant Healthcare, Inc., USA) and a third probe inserted into the outer ear canal for tympanic temperature recording (ELan Med GmbH, Germany). Temperature measurements at other sites such as the esophagus or bladder, i.e., more invasive methods were avoided in our group of healthy volunteers.

Neck skin, tympanic, and rectal temperatures were measured at baseline, immediately after cooling onset, and after every 10 min of cooling. Simultaneously, participants had to rate the perception of frostiness and overall discomfort on a visual analog scale ranging from 0 (no frostiness/discomfort) to 10 (maximum conceivable frostiness/discomfort).

After 90 min, the cooling device was removed while measurements continued for another 10 min for safety reasons and to assure return of parameters to baseline values.

Criteria to abort cooling were a decrease in systolic (BPsys) or diastolic blood pressure (BPdia) by more than 20 mmHg, bradycardia above 1200 ms (i.e., 50 bpm), tachycardia below 500 ms (i.e., 120 bpm), arrhythmias, and complaints about significant discomfort or pain indicating the participant’s desire to end the test.

### Measurement of RR intervals, blood pressure, respiration, skin blood flow, cerebral blood flow velocity, and temperatures

We continuously recorded electrocardiographic RR intervals (RRI) using a standard 3-lead electrocardiogram, and noninvasively monitored beat-to-beat BPsys and BPdia by means of radial artery applanation-tonometry at the wrist (Colin Pilot, Colin Medical) with oscillometric BP calibration at the brachial artery [12].

We recorded respiratory frequency using calibrated 2-belt chest-abdomen inductance plethysmography (Respitrace Calibrator, Ambulatory Monitoring, Inc., Ardsley, NY, USA) with 1 belt at the level of maximal thoracic and the other at maximal abdominal respiratory excursions [9].

Skin blood flow (SBF) was monitored at the right index finger pulp using laser Doppler flowmetry (Perimed, Stockholm, Sweden). The laser probe emits a divergent narrow band light at a wavelength of approximately 780 nm with an intensity of 0.8 mW [12]. The volume measured in the skin is a hemisphere with an approximate radius of 1 mm [12]. However, the instrument does not measure perfusion in absolute values (ml × min^−1^ × g^−1^), given that the measured volume is tissue-dependent and not exactly known [9, 12, 13]. Therefore, after calibration of the instrument with a motility standard according to the manufacturer, flow was measured in arbitrary perfusion units (PU) [9, 12, 13].

Mean CBFV at the right proximal MCA was recorded using transcranial Doppler ultrasonography (Multidop, DWL, Sipplingen, Germany). The MCA was insonated through the temporal window approximately 1 cm above the zygomatic arch at a depth of 35–65 mm using a pulsed 2-MHz probe. For each participant, the insonation depth was selected as that which gave the most stable and optimal signal. The probe was attached to the skull at a fixed angle using an adjustable positioning system. The CBFV of the MCA (MCA-CBFV) is proportional to CBF provided that both the angle of insonation and the diameter of the artery remain constant [9].

### Data storage and off-line analysis

RRI, BPsys, BPdia, SBF, MCA-CBFV, and respiratory frequency were sampled on a custom-designed data acquisition and analysis system (SUEmpathy™, SUESS Medizin-Technik GmbH, Aue, Germany) and stored for off-line analysis.

From 5-min recordings taken at baseline, immediately after the onset of neck cooling, after 60 min and after 90 min of neck cooling, we selected 60-s epochs without artifacts to calculate mean values and standard deviations (SD) of the above bio-signals.

### Statistical analysis

We tested data for normal distribution using the Kolmogorov-Smirnov test. Differences in RRIs, BPsys, BPdia, CBFV, SBF, and respiratory frequency values at baseline, upon cooling onset, and after 60 and 90 min of neck cooling were assessed by analysis of variance (ANOVA) for repeated measurements (general linear model). Differences in skin, rectal, and tympanic temperature values, in subjective values of frostiness and overall discomfort at baseline, upon cooling onset, and after every 10 min of cooling were also assessed by ANOVA. The suitability of the ANOVA model was determined by Mauchly’s sphericity test. In case of violation of the sphericity assumption, the Greenhouse Geisser correction was employed. In case of significant ANOVA results, we performed post hoc single comparisons. For comparison of values at baseline, prior to cooling, and during neck cooling, we used *t* tests for paired samples in case of normal distribution, and the Wilcoxon test in case of non-normal distribution of data. A commercially available statistical program (SPSS™, IBM SPSS Statistics 20, USA) was used for data analysis. Significance was set at *p* < 0.05.

## Results

### Perception of frostiness and discomfort

**Fig. 1:**
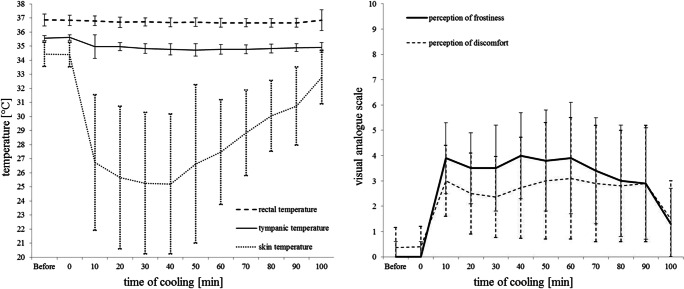
Skin, tympanic, and rectal temperature (left graph) and perception of frostiness and discomfort (right graph) before and during 90 min of neck cooling in 11 healthy participants, (presented as mean ± standard deviation)

Data are presented as mean ± SD. Visual analog scale scores reflecting the perception of frostiness (Fig. 1) remained stable from baseline to cooling onset (0.2 ± 0.6), but had already significantly increased after 10 min of cooling (3.9 ± 1.4; *p* < 0.001), and reached highest scores after 40 min of cooling (4.0 ± 1.7; *p* < 0.001). Then, frostiness scores steadily decreased until the end of cooling (3.9 ± 2.2 after 60 min; 2.9 ± 2.2 after 90 min of cooling). However, frostiness scores after 90 min of cooling were still significantly higher than scores at baseline (*p* = 0.003).

Scores of discomfort perception (Fig. 1) showed changes upon cooling that were similar to those of frostiness scores: discomfort scores also remained stable during baseline until cooling onset (0.4 ± 0.8) but significantly increased to discomfort scores of 3.0 ± 1.4 (*p* < 0.001), assessed already 10 min after cooling onset. Discomfort scores were highest after 60 min of cooling (3.1 ± 2.4; *p* = 0.002). Then, discomfort scores decreased until the end of cooling (2.9 ± 2.3 after 90 min cooling) but were still higher than scores at baseline (*p* = 0.002). The participants’ main discomfort complaint was the requirement to maintain a lying position without moving for 90 min. Despite the perception of frostiness and discomfort, all participants completed the entire study protocol with 90 min of cooling.

During neck cooling, none of our participants showed or reported shivering.

### Skin, tympanic, and rectal temperatures

Neck cooling significantly decreased skin temperature at the neck but only very slightly, albeit still significantly lowered tympanic and rectal temperatures (Fig. 1; Table 1).

**Table 1:** Rectal, tympanic, and skin temperature at baseline and lowest values during neck cooling in 11 healthy participants

	Rectal temperature at baseline (°C)	Rectal temperature lowest values (60 min cooling) (°C)	Tympanic temperature at baseline (°C)	Tympanic temperature lowest values (50 min cooling) (°C)	Skin temperature at baseline (°C)	Skin temperature lowest values (40 min cooling) (°C)
Participant #1	37.2	37.1	35.5	34.4	33.6	27.3
Participant #2	37.1	36.7	35.7	34.5	34.4	16.2
Participant #3	36.0	36.3	35.6	35.0	36.6	36.2
Participant #4	36.9	36.3	35.5	35.0	34.3	27.9
Participant #5	36.7	36.6	35.7	34.9	34.7	24.7
Participant #6	37.5	37.2	35.6	35.3	33.2	21.2
Participant #7	37.0	36.6	35.3	34.5	34.8	27.7
Participant #8	36.6	36.6	35.3	33.7	33.9	25.7
Participant #9	36.4	36.6	35.7	34.9	34.4	24.2
Participant #10	37.2	36.9	35.8	35.1	34.3	23.0
Participant #11	36.8	36.3	35.9	35.0	34.4	23.1
Mean ± SD	36.9 ± 0.4	36.7 ± 0.3*	35.6 ± 0.2	34.7 ± 0.4*	34.4 ± 0.9	25.2 ± 5.0*

*Indicates significant differences between baseline values and lowest values during neck cooling

*min* minutes, *SD* standard deviation

Neck skin temperature was stable during baseline (34.4 ± 0.9 °C) until cooling onset, but significantly decreased to 26.7 ± 4.8 °C already after 10 min of neck cooling and was lowest after 40-min cooling (25.2 ± 5.0 °C; *p* < 0.001). Then, neck skin temperature slowly re-increased until the end of cooling (27.5 ± 3.7 °C after 60 min, 30.0 ± 2.8 °C after 90 min cooling; Fig. 1; Table 1).

Tympanic temperature was also stable during baseline (35.6 ± 0.2 °C) until cooling onset. Within the first 10 min of cooling, tympanic temperature showed a very slight decrease to 35.0 ± 0.8 °C, which however, was still significant (*p* = 0.026). Tympanic temperature continued to slightly decrease and reached its nadir (34.7 ± 0.4 °C) after 50-min cooling; again, the rather small difference to baseline values was significant (*p* < 0.001). Tympanic temperature remained at similar levels after 60 min (34.8 ± 0.4 °C) and 90 min (34.9 ± 0.3 °C) of cooling (Fig. 1; Table 1).

Rectal temperature also showed only small changes during cooling. It decreased very little from baseline values of 36.9 ± 0.4 °C to 36.7 ± 0.3 °C after 60 min of cooling; still, data differed statistically (*p* = 0.043). After 60 min cooling, rectal temperature slightly re-increased and no longer differed significantly after 90 min of cooling from rectal temperature at baseline (*p* > 0.05; Fig. 1; Table 1).

### Heart rate, blood pressure, skin blood flow, respiration, and cerebral blood flow velocities

**Fig. 2:**
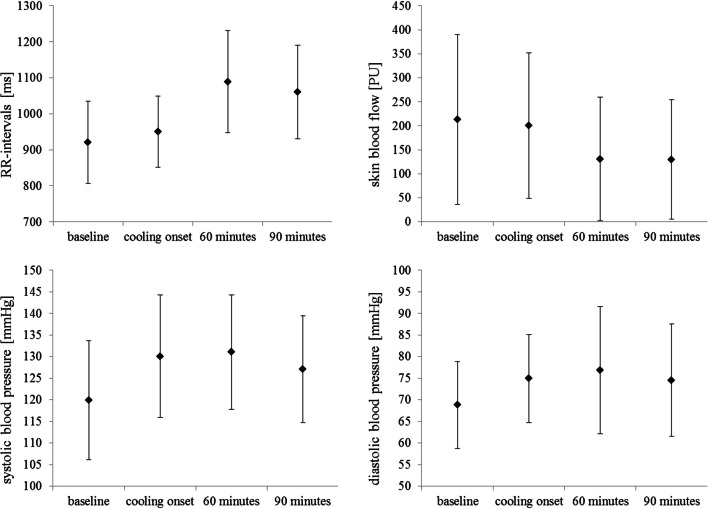
RR intervals (RRI; upper left graph), skin blood flow at the right index finger pulp (SBF; upper right graph), systolic blood pressure (BPsys; lower left graph) and diastolic blood pressure (BPdia; lower right graph) during baseline, after cooling onset, after 60 and 90 min of neck cooling in 11 healthy participants, (presented as mean ± standard deviation)

During neck cooling, HR slowed significantly: after 60-min cooling RRIs had increased from 921.0 ± 114.4 ms at baseline to 1089.1 ± 141.7 ms (*p* < 0.001). After 90 min of cooling, RRIs had slightly decreased to 1060.4 ± 130.0 ms, i.e., HR had slightly re-accelerated. However, RRIs were still higher, i.e., HR was slower at the end of cooling than at baseline (*p* < 0.001; Fig. 2; Table 2).

**Table 2:** RR intervals, systolic and diastolic blood pressure, skin blood flow at the right index finger pulp, and cerebral blood flow velocity at the middle cerebral artery during baseline, after cooling onset, after 60 and 90 min of neck cooling in 11 healthy participants. Data are presented as mean ± SD

	Baseline	Cooling onset	60 min	90 min	Baseline vs. cooling onset	Baseline vs. 60 min	Baseline vs. 90 min
RRI (ms)	921.0 ± 114.4	949.9 ± 98.8	1089.1 ± 141.7*	1060.4 ± 130.0*	*p* = 0.053	*p* < 0.001	*p* < 0.001
BPsys (mmHg)	119.9 ± 13.8	130.1 ± 14.2*	131.0 ± 13.3*	127.1 ± 12.4	*p* < 0.001	*p* = 0.018	*p* = 0.111
BPdia (mmHg)	68.8 ± 10.0	74.9 ± 10.2*	76.9 ± 14.7*	74.6 ± 13.0*	*p* = 0.008	*p* = 0.024	*p* = 0.043
SBF right index finger (PU)	213.2 ± 177.0	200.5 ± 151.3	130.7 ± 128.7*	129.9 ± 124.8*	*p* = 0.594	*p* = 0.003	*p* = 0.006
CBFV (cm s^−1^)	77.3 ± 27.4	78.7 ± 25.8	78.9 ± 28.1	78.5 ± 27.8	*p* = 0.610	*p* = 0.481	*p* = 0.565

*Indicates significant differences between baseline values and values after cooling onset and after 60 and 90 min of neck cooling

*RRI* RR intervals, *BPsys* systolic blood pressure, *BPdia* diastolic blood pressure, *SBF* skin blood flow, *CBFV* cerebral blood flow velocity, *min* minutes

After the onset of neck cooling, BPsys significantly increased within the first 5-min of cooling from 119.9 ± 13.8 mmHg at baseline to 130.1 ± 14.2 mmHg, and to 131.0 ± 13.3 mmHg after 60-min cooling. Then, BPsys slightly decreased to 127.1 ± 12.4 mmHg and no longer differed from baseline values after 90-min cooling (*p* > 0.05; Fig. 2; Table 2).

Similarly, BPdia increased steadily and significantly from 68.8 ± 10.0 mmHg at baseline to 74.9 ± 10.2 mmHg within the first 5 min upon cooling onset, and to 76.9 ± 14.8 mmHg after 60 min of cooling. After 90-min cooling, BPdia was at 74.6 ± 13.0 mmHg, i.e., BPdia values were still higher than at baseline (*p* = 0.043; Fig. 2; Table 2).

Index finger SBF was stable during baseline (213.2 ± 177.0 PU) until cooling onset (200.5 ± 151.3 PU) but decreased significantly during cooling to values of 130.7 ± 128.7 PU after 60-min cooling and 129.9 ± 124.8 PU after 90 min of cooling (Fig. 2; Table 2).

Respiratory frequency and MCA-CBFV remained unchanged during neck cooling (*p* > 0.05; Table 2).

## Discussion/conclusion

In our non-sedated healthy participants, isolated neck cooling significantly lowered skin temperature at the neck and minimally although still significantly lowered rectal, and tympanic temperatures. At the time when the average decreases were most prominent for all participants, the individual changes ranged between + 0.3 °C and − 0.6 °C for rectal temperature and between − 0.1 °C and − 1.6 °C for tympanic temperature (Table 1).

In contrast, the prominent decrease in skin temperature from 34.4 ± 0.9 °C to 25.2 ± 5.0 °C was perceived as quite uncomfortable and was associated with significant cardiovascular effects. Immediately upon cooling onset, the participants scored discomfort around 3/10 throughout the 90-min cooling period (Fig. 1). Their perception of frostiness was even higher and reached 4/10 at the first evaluation, after 10 min of cooling. Perception of frostiness only decreased after 60 min of cooling but did not attenuate below 3/10 by the end of cooling (Fig. 1).

Cardiovascular responses to neck cooling were similar to those seen during cold face stimulation [14, 15] although the facial area was spared from cooling during this study.

In our non-sedated participants, the unpleasant cold-stimulus immediately induced a significant increase in BPsys from 119.9 ± 13.8 mmHg to 130.1 ± 14.2 and in BPdia from 68.8 ± 10.0 mmHg to 74.9 ± 10.2 mmHg upon cooling onset, and continued cold perception kept BPsys and BPdia at similar levels throughout the entire cooling period. The BP increase was due to sympathetically mediated peripheral vasoconstriction as shown by the decrease in superficial skin perfusion at the index finger pulp, an area exclusively innervated by sympathetic vasomotor fibers [8, 16]. Index finger SBF dropped from 213.2 ± 177.0 PU prior to cooling to only 130.7 ± 128.7 PU after 60 min and remained at this level until the end of cooling. Peripheral vasoconstriction is one of the primary autonomic defenses against cold and buffers core-to-periphery heat transfer and heat loss [17]. Furthermore, shivering can transiently increase metabolic rates which might increase local temperature and thus limit therapeutic hypothermia in non-sedated persons [18]. However, in our study, none of the participants showed or reported shivering during neck cooling.

While the continuous peripheral vasoconstriction explains the rise in BPsys by approximately 10 mmHg and in BPdia by approximately 6–7 mmHg throughout the cooling period, it was somewhat unexpected that HR slowed significantly as evidenced by the increase in RRIs during the 90-min neck cooling (Fig. 2 Table 2). Usually, cold stimulation of the skin that spares the facial area induces sympathetically mediated BP increase and HR acceleration [8, 19, 20]. In contrast to this “cold pressor response,” our participants’ HR slowing in the presence of BP increase was similar to the responses seen with cold face stimulation [8, 15] although we performed isolated neck cooling specifically to avoid cooling of the skin innervated by the trigeminal nerve.

There might be three potential explanations for the slowing of HR. Either, there are nervous anastomoses from sensory fibers of the cervical plexus, particularly the occipitalis minor, transversus colli, auricularis magnus, and even supraclavicular nerves to the trigeminal facial fibers [21] that still activated trigeminal impulses which are considered to account for the HR slowing during a cold face test [8, 15], or neck cooling was so intense that thermal convection still cooled facial skin areas and thus triggered HR slowing. However, it seems more likely that the HR slowing in our participants was due to continuous baroreflex activation by the enduring, cold-induced BP increase that resulted in baroreflex-mediated cardiovagal activation with subsequent HR slowing. We assume that the cardiovagal baroreflex response overrode any cold-induced, baroreflex-independent sympathetic activation and HR acceleration as it occurs during short-lasting cold pressor stimulation [20].

Despite the significant BP increase, MCA-CBFV did not change during the 90-min cooling period. This result is to be expected in young healthy persons who have normal cerebral autoregulation that adequately buffers any change in BP within the limits of healthy cerebral autoregulation, i.e., within a mean BP range from 50 to 170 mmHg [8, 22]. In one of our previous studies, Brown et al. found an increase in cerebrovascular resistance during 1-min cold stimulation and concluded that there is constriction of cerebral resistance vessels upon sympathetic activation [9]. Thus, the sympathetically mediated increase in cerebrovascular resistance would buffer the effects of the sympathetically mediated BP increase, and consequently assures stable CBFV in conjunction with hormonal and metabolic factors of cerebral autoregulation [9].

However, cerebral autoregulation may be compromised in patients who suffered an acute brain injury, particularly a stroke or subarachnoid hemorrhage [23, 24]. Therefore, it is unclear how CBFV would change in patients in response to a 10 mmHg BP increase. Cerebral autoregulation might fail to buffer increased BP levels which could further damage injured brain tissue, e.g., by hemorrhagic transformation [25], or could destroy penumbra tissue at risk [25]. Moreover, acute stroke is associated with an increase in sympathetic activity [26] which could further augment the BP increase induced by neck cooling. According to clinical trials, even variations in peak BPsys are associated with an increased hazard ratio of parenchymal hemorrhages after ischemic stroke [27]. Furthermore, intravenous thrombolysis is only recommended for BP levels below 180/105 mmHg [28]. Therefore, any intervention triggering sympathetic activation with vasoconstriction and arterial hypertension, such as neck cooling, may be harmful and may therefore limit the applicability of revascularization treatment.

This study was limited by a lack of comparisons with a target population such as patients with ischemic stroke. Yet, we first needed to evaluate whether the procedure might have negative effects before applying neck cooling in clinical scenarios. In contrast to our healthy study participants, sympathetic responses to neck cooling might be even more prominent in older stroke patients. With increasing age, there is a shift towards augmented sympathetic and reduced parasympathetic cardiovascular modulation [26, 29–31]. After acute stroke, patients have an even more prominent increase in sympathetic cardiovascular modulation while cardiovagal modulation is significantly reduced [26, 29–31]. Given the findings in our healthy volunteers, we are concerned that further studies evaluating physiological regulatory mechanisms of neck cooling in stroke patients may be harmful and might expose patients to unpredictable risk.

Probably, the significant BP increase induced by neck cooling might be attenuated by sedating and pain-relieving medication [1]. In patients undergoing invasive cooling, sedation or general anesthesia may prevent harmful BP increases [1]. General anesthesia inhibits the cooling induced peripheral vasoconstriction that normally serves as thermoregulatory defense against the loss in core temperature [18]. However, sedating medication compromises the correct assessment of the patient’s status and level of consciousness, i.e., of parameters that are essential for further therapeutic decisions in acute stroke patients or patients with other acute brain injuries [32]. Therefore, sedation of these patients should be considered with care, particularly during the prehospital phase.

Most importantly, isolated neck cooling not only increased BP significantly and lowered HR but also had very limited if not negligible effects on core and tympanic temperatures and therefore quite likely on intracranial temperature. Since we studied neck cooling effects in healthy individuals, we were not able to invasively monitor actual brain temperature but had to rely on tympanic temperature which might to some extent reflect intracranial temperature [1]. After 50-min cooling, tympanic temperature decreased by 0.8 ± 0.4 °C which might suggest that there could have been some effect on the cerebral temperature (Fig. 1) [1]. Yet, we cannot rule out that cold conduction from the skin to the outer ear region contributed to the decrease in tympanic temperature.

Finally, recent studies suggest that hypothermia does not have major beneficial effects in the clinical setting [33, 34]. Although several studies suggested that hypothermia might have neuroprotective benefits in patients with ischemic tissue injury [1], recent trials—e.g., the POLAR-RCT—showed that prophylactic hypothermia has only limited effects and is associated with increased rates of adverse events [33]. Furthermore, Neugebauer et al. recently showed in patients with malignant MCA stroke that moderate hypothermia early after hemicraniectomy did not improve mortality rates or functional outcome compared to hemicraniectomy treatment only [34].

In conclusion, we found a prominent decrease in skin temperature and a statistically significant though minor and therefore clinically questionable decrease in tympanic temperature. Our current results cannot provide a final answer to the question whether neck cooling lowers cerebral temperature. To determine whether noninvasive neck cooling might be suitable to induce efficient brain cooling, more direct measurements of intracerebral temperature would be required. Yet, such measurements require an invasive approach which must not be applied in healthy volunteers, and might at best be considered under most stringent monitoring of potentially harmful cardiovascular changes in a well-selected group of patients, e.g., patients who have low–normal BP. Instead, our current results indicate that any assumed neuroprotective effect of potentially lowered cerebral temperature may be outweighed by the neck cooling induced BP increase seen in non-sedated persons. In summary, isolated neck cooling does not seem to be suited for hypothermia induction in non-sedated patients since its intracranial cooling effects seem to be small if not absent and are therefore inferior to those of systemic, invasive cooling.
