# Correction to: Neck cooling induces blood pressure increase and peripheral vasoconstriction in healthy persons

**DOI:** 10.1007/s10072-021-05355-3

**Published:** 2021-07-05

**Authors:** Julia Koehn, Ruihao Wang, Carmen de Rojas Leal, Bernd Kallmünzer, Klemens Winder, Martin Köhrmann, Rainer Kollmar, Stefan Schwab, Max J. Hilz

**Affiliations:** 1 Department of Neurology, University of Erlangen-Nuremberg, Schwabachanlage 6, 91054 Erlangen, Germany; 2 Department of Neurology, Universitätsklinikum Essen, Hufelandstr. 55, 45147 Essen, Germany; 3 Department of Neurology, General Hospital Darmstadt, Grafenstr. 9, 64283 Darmstadt, Germany; 4 Department of Neurology, Icahn School of Medicine at Mount Sinai, New York, NY USA

**Correction to: Neurological Sciences (2020) 41:2521–2529**

**https://doi.org/10.1007/s10072-020-04349-x**

The article “Neck cooling induces blood pressure increase and peripheral vasoconstriction in healthy persons”, written by Julia Koehn, Ruihao Wang, Carmen de Rojas Leal, Bernd Kallmünzer, Klemens Winder, Martin Köhrmann, Rainer Kollmar, Stefan Schwab and Max J. Hilz, was originally published Online First without Open Access. After publication in volume 41, issue 9, page 2521–2529 the authors decided to opt for Open Choice and to make the article an Open Access publication. Therefore, the copyright of the article has been changed to © The Author(s) 2020 and the article is forthwith distributed under the terms of the Creative Commons Attribution 4.0 International License, which permits use, sharing, adaptation, distribution and reproduction in any medium or format, as long as you give appropriate credit to the original author(s) and the source, provide a link to the Creative Commons licence, and indicate if changes were made. The images or other third party material in this article are included in the article’s Creative Commons licence, unless indicated otherwise in a credit line to the material. If material is not included in the article’s Creative Commons licence and your intended use is not permitted by statutory regulation or exceeds the permitted use, you will need to obtain permission directly from the copyright holder. To view a copy of this licence, visit http://creativecommons.org/licenses/by/4.0.

The original article has been corrected.

